# Feeding time

**DOI:** 10.7554/eLife.08166

**Published:** 2015-05-27

**Authors:** Hugh D Piggins, David A Bechtold

**Affiliations:** Faculty of Life Sciences, University of Manchester, Manchester, United Kingdomhugh.d.piggins@manchester.ac.uk; Faculty of Life Sciences, University of Manchester, Manchester, United Kingdomdavid.bechtold@manchester.ac.uk

**Keywords:** circadian clock, liver, food resetting, clock genes, per, mouse

## Abstract

A hormone released from the gut after a meal can reset clock gene activity in the liver.

**Related research article** Landgraf D, Tsang AH, Leliavski A, Koch CE, Barclay JL, Drucker DJ, Oster H. 2015. Oxyntomodulin regulates resetting of the liver circadian clock by food. *eLife* **4**:e06253. doi: 10.7554/eLife.06253

**Image** When and what we eat can reset our body clocks

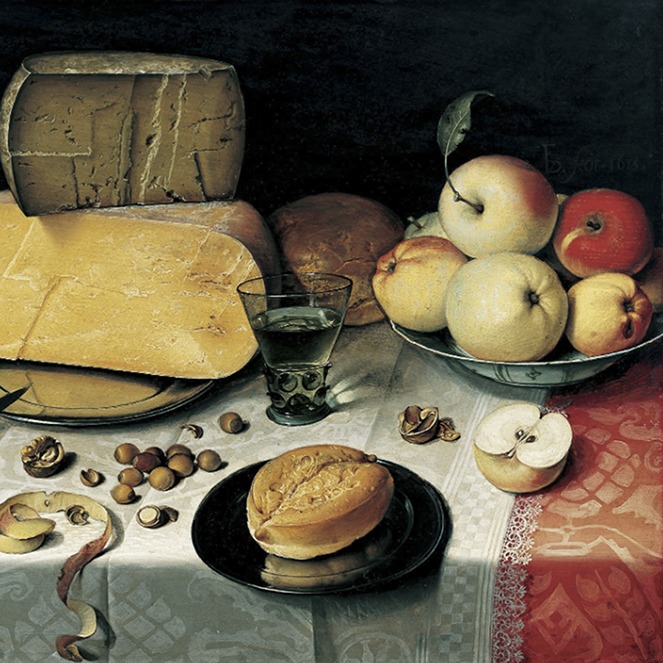

All forms of life have to be able to cope with changes in their environment, including daily cycles in temperature and light levels. As a result, organisms as diverse as bacteria and humans have evolved inbuilt timekeeping mechanisms that are capable of tracking the 24-hour day.

These so-called ‘circadian’ clocks enable organisms to anticipate changes that take place in their environment, and adapt their biology accordingly. In addition to tracking time, biological clocks must stay synchronized (or entrained) with the world around them. To achieve this, circadian clocks can be influenced or reset by environmental factors called ‘zeitgebers’ (from the German for ‘time-giver’). The daily cycle of light and dark is a dominant zeitgeber for most organisms, while periodic food availability represents another powerful zeitgeber. Now, in *eLife*, Henrik Oster and colleagues—including Dominic Landgraf and Anthony Tsang as joint first authors—report how a hormone released from the gut after eating can help the body to track changes in mealtimes (Landgraf et al., 2015).

Circadian clocks have a profound impact on mammalian biology, and virtually all aspects of our lives—from sleep-wake cycles to patterns of hormone release and energy metabolism—follow pronounced daily rhythms (Albrecht, 2012). In mammals, it is well established that the suprachiasmatic nucleus (or SCN) contains the body's master clock. Located in the brain, just above the optic nerves, the SCN clock receives information about environmental light levels directly from the eyes, which keeps it in sync with the external world.

Twenty years of research into circadian clockwork mean that we understand relatively well how changes in light adjust the timing of the SCN clock. The expression of specific ‘clock genes’, such as *Per1* and *Per2*, within neurons of the SCN is increased in response to light. This means that the SCN clock can be advanced or delayed to ensure it remains in time with the prevailing light–dark cycle. However, it has also become clear that there are other circadian clocks in most of the cells and tissues in the body (Guilding and Piggins, 2007; Mohawk et al., 2012).

Under normal circumstances, this network of clocks is kept in synchrony by the master clock in the SCN; but it has been known for many decades that behavioural rhythms in laboratory rodents can be entrained by restricting access to food to certain times of the day. These food-entrained rhythms are not affected by the light–dark cycle, and they can persist in animals that have had their SCN destroyed. A set mealtime is now known to be a dominant zeitgeber for these peripheral tissue clocks (such as the clock in the liver), with the expression of the clock genes in these tissues become aligned to feeding time (Damiola et al., 2000). In contrast, the SCN clock remains locked to the light–dark cycle. It makes sense for tissue-specific clocks to be sensitive to food instead of light because the availability of food in nature may not always coincide with other environmental factors.

Unlike modulation of the SCN clock by light, it is unclear which signals convey information about feeding time to reset the circadian clock in the liver. Oster, Landgraf, Tsang and colleagues—who are based at the Max Planck Institute for Biophysical Chemistry, and the Universities of Lübeck and Toronto—report that a gut hormone called oxyntomodulin is one of these signals. Oxyntomodulin is a peptide hormone that is released from the gut in response to food intake, and has been suggested to be a potential drug target to combat obesity in humans (Druce and Bloom, 2006).

Landgraf, Tsang et al. started by screening around 200 peptide molecules that are known to be involved in appetite and the regulation of body weight to see if any could adjust the molecular clock of liver tissue. This in vitro screen identified two molecules: oxyntomodulin and glucagon. In particular, treatment with oxyntomodulin could shift the liver clock by several hours, either forward or back, depending on the time it was administered. Both of these characteristics suggest that oxyntomodulin serves to set the liver clock to feeding rhythms in living organisms.

So how does oxyntomodulin reset the liver clock? Somewhat unusually, this hormone can bind to two different types of receptor protein (namely GLP-1 receptors and glucagon receptors; Pocai, 2013). Further experiments confirmed that oxyntomodulin's liver resetting activity depends on it stimulating glucagon receptors (and not GLP-1 receptors). This stimulation leads to a transient increase in the expression of the *Per1* clock gene in the liver cells, which involves a signalling cascade that is reminiscent of the light-induced responses of the SCN (Tischkau et al., 2003).

Mice that were given oxyntomodulin when they would normally be resting showed an increase in *Per* expression in the liver, and experienced a shift in the timing of their liver clock. Treatment with oxyntomodulin also delayed and/or reduced the expression of genes involved in carbohydrate metabolism. However, treating the mice with oxyntomodulin during the period when they were normally active had no effect on the liver. Thus, oxyntomodulin's effects on the liver clock only occur at times of the daily cycle when the mice do not typically eat. Furthermore, oxyntomodulin had no effect the SCN master clock.

These experiments only demonstrate that artificially elevated levels of oxyntomodulin alter liver activity, so Landgraf, Tsang et al. then explored whether a mouse's normal levels of oxyntomodulin act to regulate its liver clock. When fasted animals were then given the opportunity to eat, oxyntomodulin levels in the blood increased within 20 minutes, and remained high for one hour. This signal was enough to alter clock gene activity in the liver. Landgraf, Tsang et al. then treated mice with antibodies that bind to oxyntomodulin. This treatment neutralized the hormone circulating in the body, and prevented its action, which weakened the resetting of the liver clock following food intake.

Landgraf, Tsang et al. suggest that the oxynto-modulin released by the gut following a meal serves as a timing cue for the liver clock. When food intake occurs at the expected times of day, the liver clock is relatively blind to this signal. However, when a meal occurs outside of a normal feeding time, the induced rise in oxyntomodulin serves to adjust the clock to a new feeding schedule. In this way, oxyntomodulin may function as an important signal that translates feeding time into the timing of an internal clock.

It will be important to examine oxyntomodulin signalling in situations were food is limited, and to explore whether it can also shift the clocks in other peripheral tissues, such as muscles and the pancreas. Furthermore, the therapeutic potential of this peptide hormone in correcting problems associated with ‘jet-lag’ and rapid travel through multiple time zones also awaits investigation.
